# Navigating the Diagnostic and Management Challenges of an Anterior Mediastinal Tumor: A Case Report

**DOI:** 10.7759/cureus.70511

**Published:** 2024-09-30

**Authors:** Vaishnavi Reddy, Parin Patel, Manoj M Dongare, Ankit Maheshwari, Dakshayani S Nirhale

**Affiliations:** 1 General Surgery, Dr. D. Y. Patil Medical College, Hospital and Research Centre, Dr. D. Y. Patil Vidyapeeth, Pune, IND; 2 Hepatico-Pancreatic Billiary and Transplant Surgery, Dr. D. Y. Patil Medical College, Hospital and Research Centre, Dr. D. Y. Patil Vidyapeeth, Pune, IND; 3 Cardiothoracic Surgery, Dr. D. Y. Patil Medical College, Hospital and Research Centre, Dr. D. Y. Patil Vidyapeeth, Pune, IND

**Keywords:** anterior mediastinum, mature type teratoma, mediastinal teratomas, thymus, total median sternotomy

## Abstract

Mediastinal teratomas, originating from pluripotent embryonic cells, are uncommon germ cell tumors that contain tissues from all three germ layers. Despite being the most frequent germ cell tumors in the mediastinum, they remain rare overall. This case describes a 19-year-old male who presented with chest pain, shortness of breath, and difficulty in swallowing and was ultimately diagnosed with a mature cystic teratoma in the anterior mediastinum. Imaging and histopathological analysis confirmed a large cystic teratoma, which was successfully removed via median sternotomy. Although the postoperative period was complicated by air leaks, infections, and an extended hospital stay, the patient fully recovered and was symptom-free at the one-month follow-up. This case underscores the value of comprehensive diagnostic assessment and demonstrates the favorable prognosis associated with complete surgical removal of thymic teratomas.

## Introduction

Mediastinal teratomas are a type of germ cell tumor (GCT) that arise from pluripotent embryonic cells, which possess the ability to differentiate into tissues containing all three germ layers: ectoderm, mesoderm, and endoderm. These tumors can contain a variety of tissue types, such as hair, muscle, or even teeth, reflecting their embryonic origin. Teratomas are generally congenital and occur in approximately one in every 4,000 live births [1]. However, mature mediastinal teratomas are relatively rare compared to other locations such as the sacrococcygeal region, retroperitoneum, or gonads, where the majority of GCTs are found. 

Among the mediastinal tumors, teratomas are the most common GCTs but still represent only about 8% of all mediastinal masses [1]. The mediastinum is an area in the chest that contains the heart, thymus, portions of the esophagus, and major blood vessels. Tumors in this region, although uncommon, can cause significant clinical concern due to their proximity to vital structures.

Thymic cysts associated with mature mediastinal teratomas are rare and present unique challenges in clinical diagnosis and management. Thymomas, which arise from the epithelial cells of the thymus, account for around 20% of anterior mediastinal tumors in adults but are infrequent in children. They typically affect individuals between the ages of 40 and 60 years and can vary in their degree of malignancy [2]. Differentiating between thymomas and teratomas is essential, as their treatment strategies and prognoses differ significantly.

Diagnosis of mediastinal masses often requires a combination of imaging studies and histopathological confirmation. Chest X-rays and computed tomography (CT) scans are frequently used to assess the location, size, and composition of the tumor. Biopsy and immunohistochemistry (IHC) testing provide further detail by identifying cellular markers that differentiate thymomas from teratomas.

## Case presentation

A 19-year-old man presented with a 15-day history of progressively worsening chest pain and shortness of breath. The symptoms were exacerbated when lying down but improved upon sitting upright, indicating a possible mechanical issue related to posture. The patient also reported dysphagia and odynophagia, which suggested that the esophagus or mediastinum was involved in the pathology. He had no history of comorbidities or chronic illness, making the acute onset of symptoms concerning. The patient’s vitals were stable upon physical examination, but his pale complexion suggested anemia or a chronic underlying condition. Respiratory examination revealed asymmetrical chest movements, suggesting a localized pathology in the thorax. There were fewer breath sounds in the left infra-axillary and inframammary regions, raising the possibility of pleural or lung involvement. We also noted muffled heart sounds, potentially indicating fluid accumulation or mass effect in the mediastinal space. Systemic examination, including cardiovascular, gastrointestinal, and neurological assessments, revealed no other abnormalities.

Routine blood investigations, including complete blood count, liver function tests, and renal function tests, were within normal limits, ruling out systemic infections or metabolic disturbances. However, chest X-ray findings were significant, showing mediastinal widening, a sign that pointed toward a mass or mediastinal shift.

**Figure 1:**
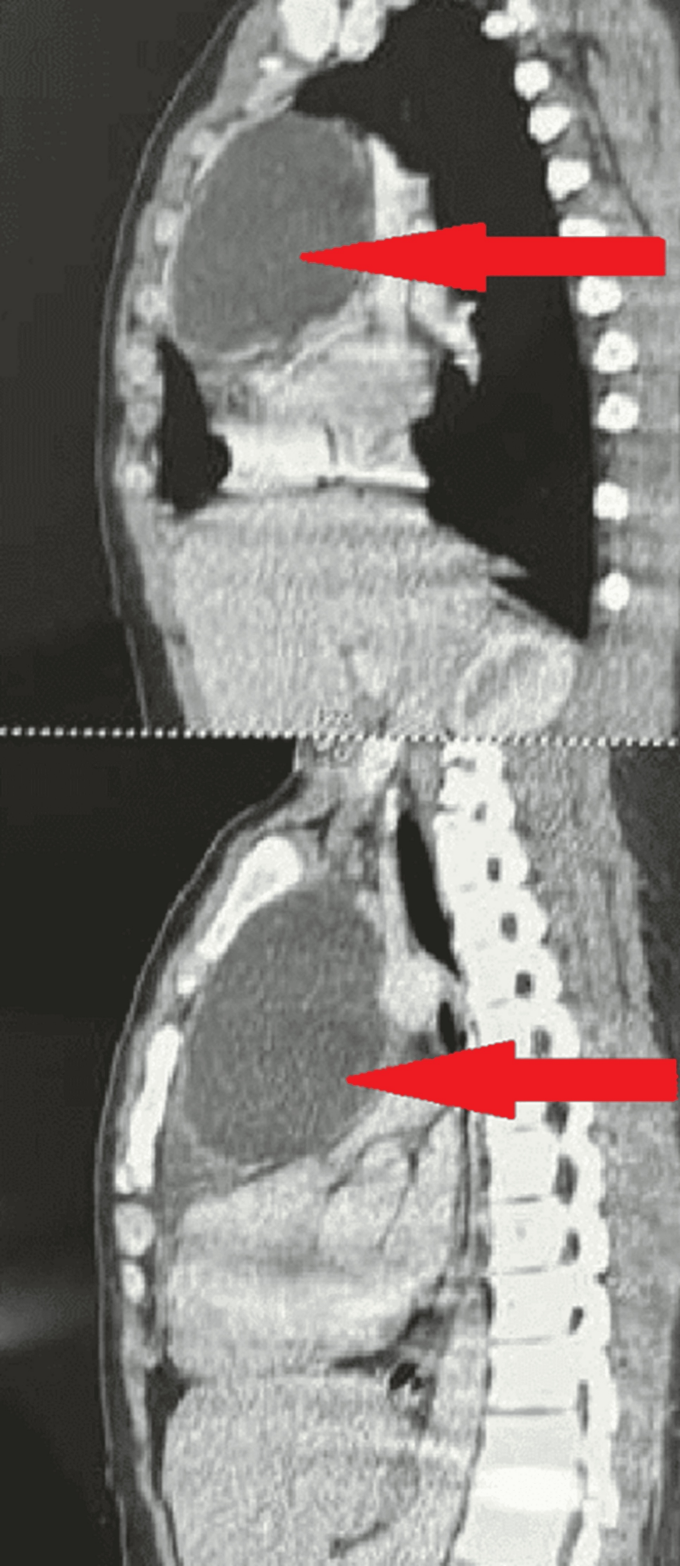
High-resolution computed tomography image of an anterior mediastinal mass

**Figure 2:**
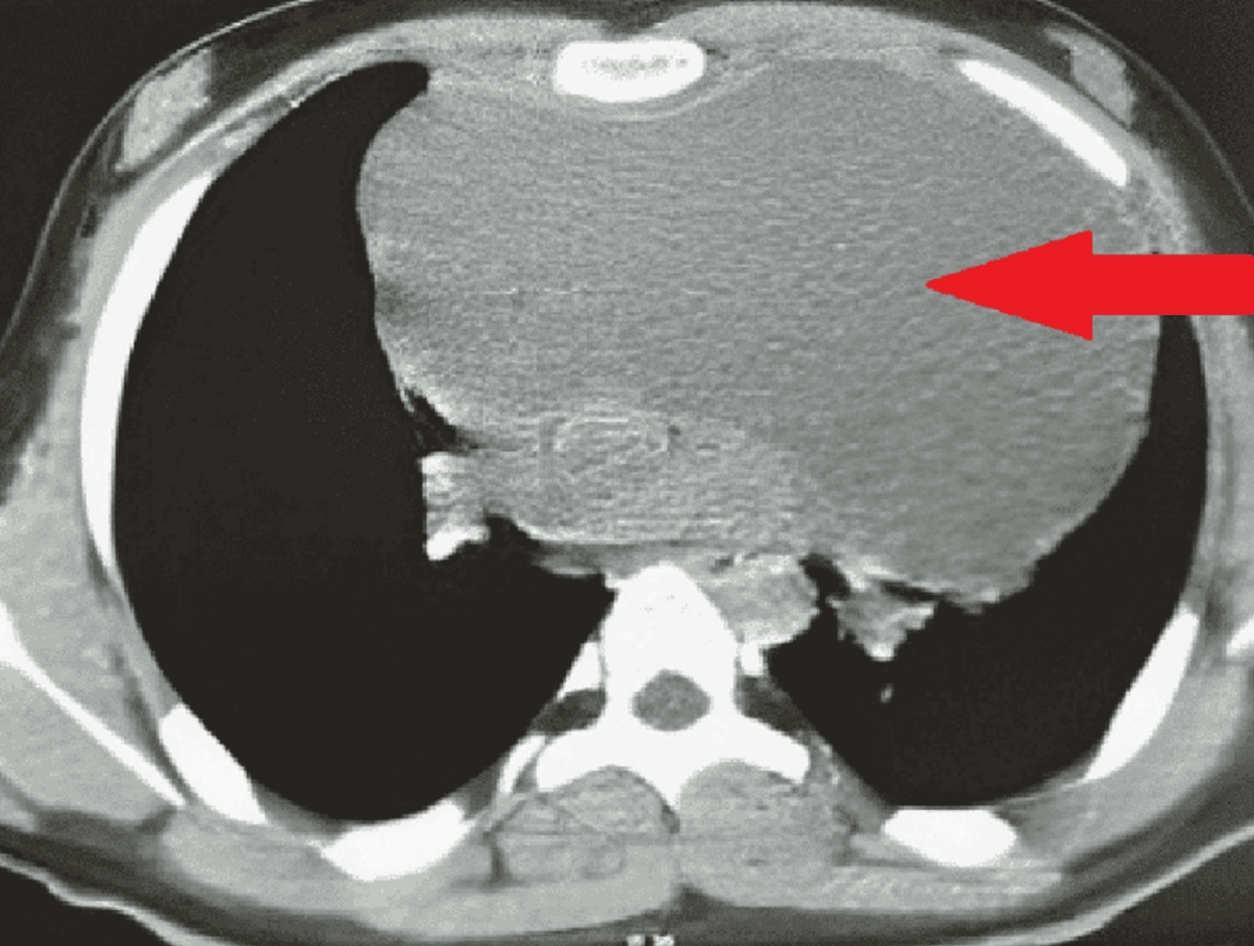
High-resolution resolution computed tomography image of anterior mediastinal well-differentiated cystic mass

High-resolution computed tomography (HRCT) of the thorax further delineated the pathology (Figures 1, 2). It revealed a large anterior mediastinal mass, approximately 15 x 20 cm in size, along with a small amount of pleural effusion on the left side. These raised concerns about a mediastinal tumor, potentially originating from the thymus or other mediastinal structures, and indicated a need for surgical intervention because of the mass’s size and potential complications.

**Figure 3:**
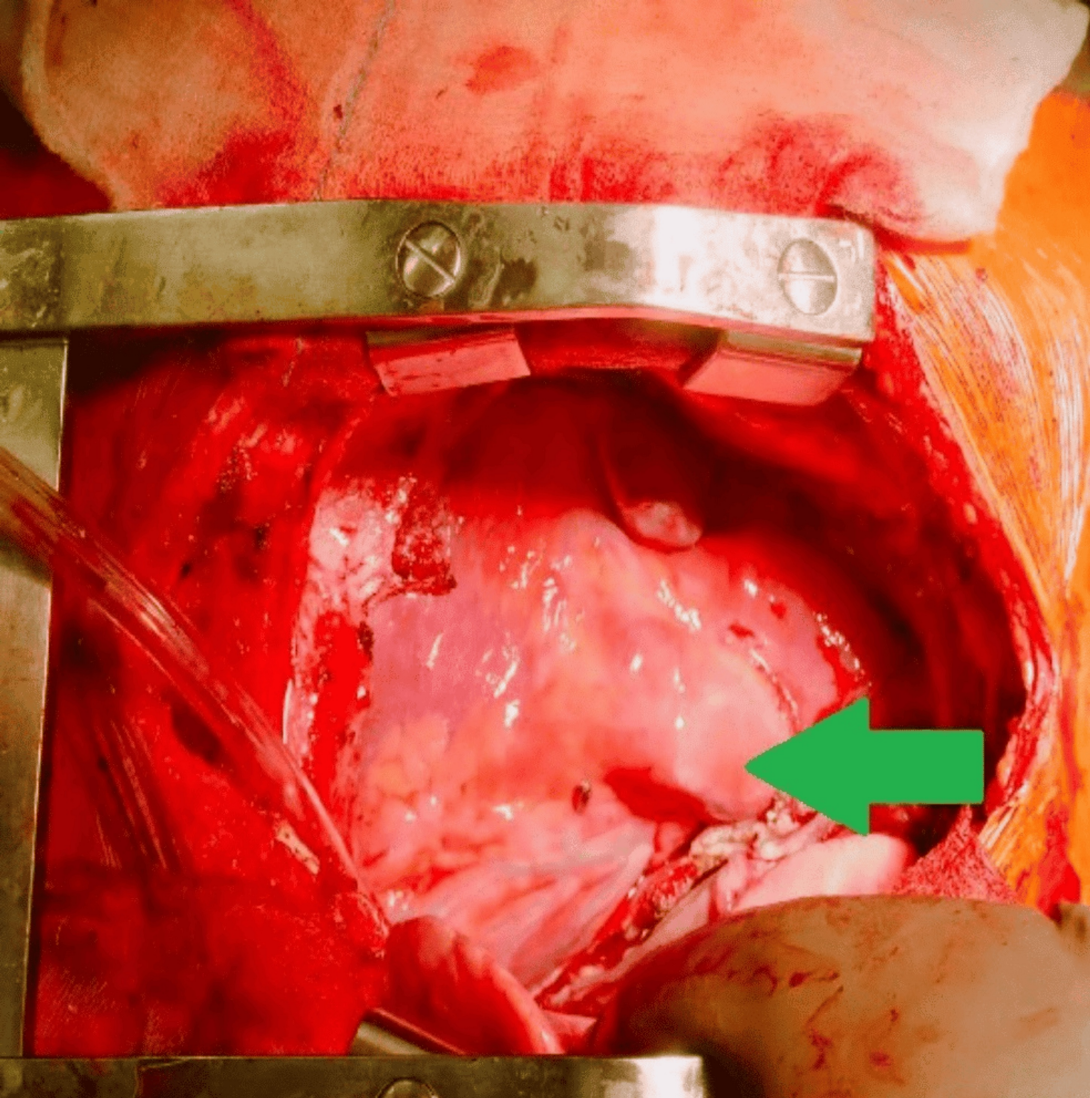
intraoperative finding of anterior mediastinal mass adherent to pericardium and pleura

**Figure 4:**
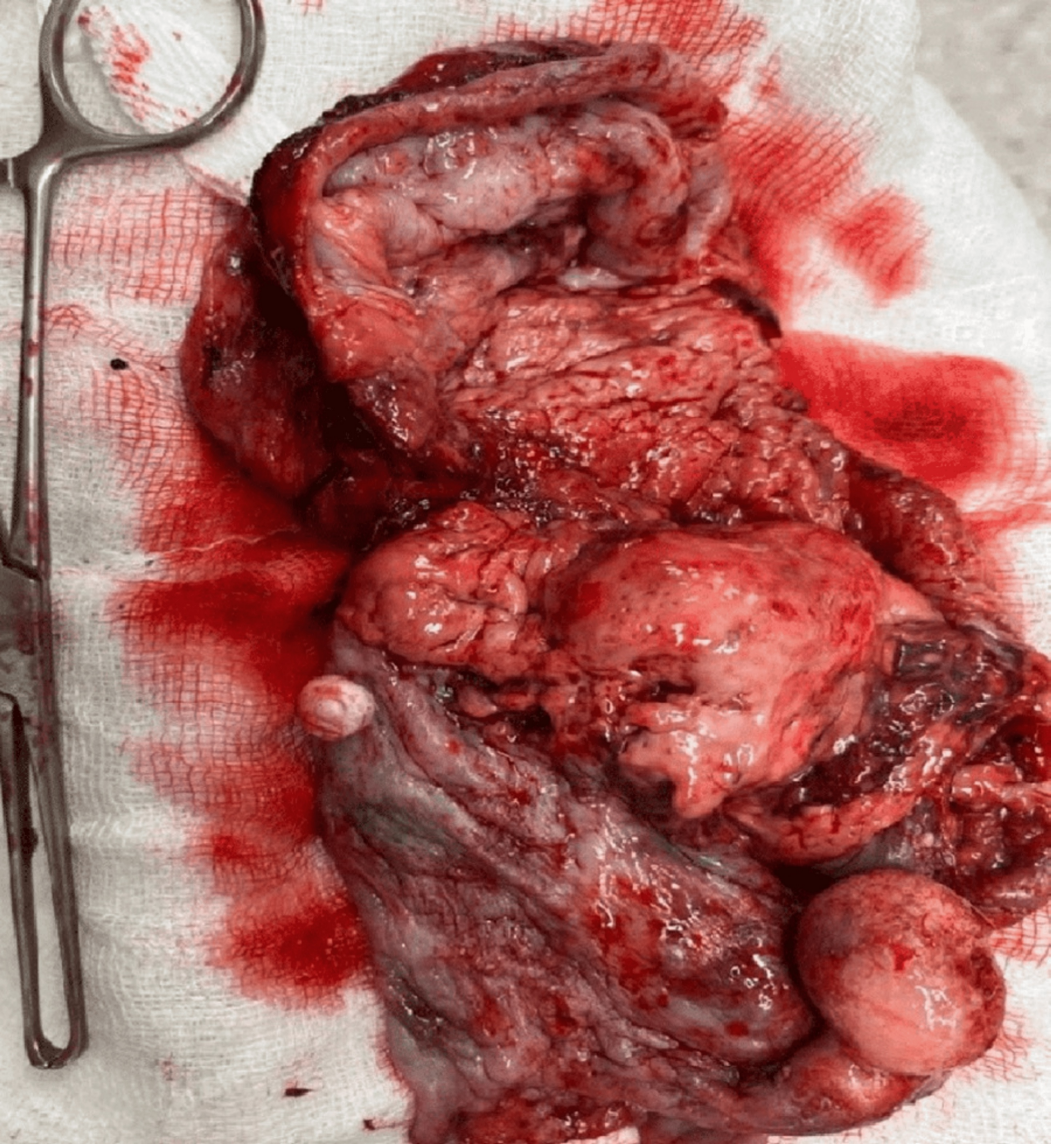
Excised specimen of mature anterior mediastinal teratoma along with anterior pericardium and parts of pleura

**Figure 5:**
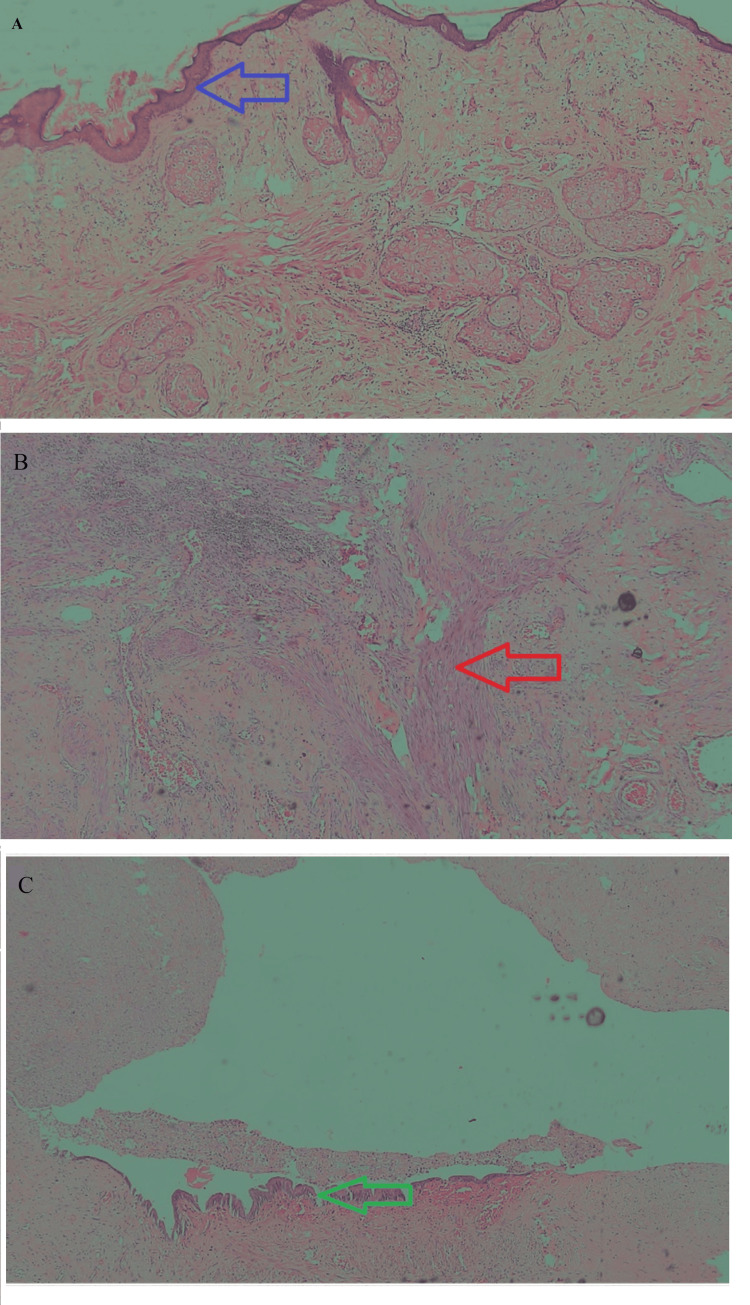
Histopathological findings suggestive of mature cystic teratoma of thymus The slides contain all three germ layers: (A) Ectodermal: squamous epithelium, sebaceous glands, hair follicles as marked with the blue arrow; (B) Mesodermal: cartilage, fibro-adipose tissue as marked with the red arrow; (C) Endodermal: columnar gastric mucosa as marked with the green arrow

Given the findings, the patient underwent an urgent median sternotomy to remove the mass. The surgeon discovered a large cystic lesion measuring 15 x 20 cm intraoperatively. The mass adhered closely to the pleura and anterior pericardium (Figure 3), but there was no evidence of invasion into the surrounding vital structures such as the heart, lungs, or great vessels. Aspirating the lesion drained approximately 300 cm of dark, foul-smelling, muddy fluid, suggesting a chronic infectious or necrotic process within the cyst. We delineated the mass and traced it back to the thymus, confirming its thymic origin. The mass was excised in toto (Figure 4) and sent for histopathological examination (Figure 5).

Following the excision, we identified an air leak from the lung parenchyma, likely due to iatrogenic injury during the procedure. We sutured the air leak using Prolene 4-0 (Ethicon, Inc., Raritan, New Jersey, United States) and placed bilateral pleural and mediastinal drains to manage any ongoing air leaks or fluid accumulation. We transferred the patient to the intensive care unit for close monitoring.

Continuous air leaks over the first three postoperative days (PODs) led to a tidal volume loss of about 120 mL. A repeat chest X-ray confirmed the presence of a pneumothorax, particularly on the right side, necessitating the placement of an intercostal drain (ICD) on the right side. We performed a bronchoscopy to further assess the air leak, revealing multiple areas of lung parenchymal injury. Surgical reintervention was deferred in favor of a conservative, wait-and-watch approach with chest drainage because of the multifocal nature of the air leaks.

On POD 4, the patient began experiencing fever spikes, and laboratory tests revealed a marked leukocytosis (WBC count 20 × 10^9^/L), suggestive of infection. Given the patient’s postoperative state and cyst fluid characteristics, we initiated broad-spectrum antibiotics, including teicoplanin and meropenem, to cover potential pathogens.

An HRCT scan on POD 7 prompted the placement of another ICD on the left side because of the reaccumulation of pleural fluid. Persistent fever and purulent drainage from the bilateral ICDs indicated a likely postoperative infection. We obtained cultures of the fluid and adjusted the antibiotic regimen based on the sensitivity results. Ultrasound-guided repositioning of the ICDs ensured optimal drainage.

The patient required intermittent continuous positive airway pressure support to maintain oxygenation because of prolonged intubation, which necessitated a tracheostomy for better airway management. The tracheostomy allowed for improved respiratory function and patient comfort during the extended recovery phase.

By POD 44, the purulent output from the ICDs had significantly decreased, enabling the gradual removal of the drains. Serial chest X-rays showed improved lung re-expansion, and the patient’s air leaks resolved over time. The patient’s oxygen saturation levels stabilized, leading to the weaning off of respiratory support. By POD 50, the patient had achieved independent breathing on room air and had closed the tracheostomy. The patient was discharged in stable condition. At the one-month follow-up, he was asymptomatic with no signs of recurrence or infection. He reported no respiratory issues, and the physical examination was unremarkable.

## Discussion

Mature cystic teratomas or dermoid cysts are benign GCTs containing mature tissues from the three germ layers: ectoderm, mesoderm, and endoderm. Although these tumors are most commonly found in the ovaries of women of reproductive age, extragonadal manifestations, such as in the mediastinum, can also occur. Thymic teratomas are an exceptionally rare entity, constituting less than 10% of all mediastinal tumors and approximately 3-12% of all teratomas [1]. GCTs in the mediastinum are predominantly malignant, with only 1-3% being benign, and benign thymic teratomas account for a mere 0.28% of all adult GCTs [1].

Although many thymic teratomas are asymptomatic, the mass effect of large lesions can cause significant clinical symptoms, including dyspnea, chest pain, cough, and, in severe cases, respiratory distress [3]. Additionally, although rare, these tumors can rupture or erode into the surrounding structures, causing complications such as pericardial or pleural effusion. In the present case, the patient reported worsening chest pain, shortness of breath, and dysphagia, symptoms likely related to the tumor’s compression of the surrounding mediastinal structures.

The diagnosis of mediastinal teratomas typically begins with a chest X-ray, which may reveal a mediastinal mass or widening. However, computed tomography (CT) is the gold standard for diagnostic evaluation. CT allows for the characterization of the teratoma, which often presents as a well-defined, lobulated cystic mass with features such as fibrofatty tissue with calcifications. Notably, more than 75% of teratomas demonstrate fatty foci, with up to 50% containing tooth- or bone-like calcifications, a characteristic feature that aids in diagnosis [2]. In recent years, magnetic resonance imaging has emerged as a complementary tool, offering enhanced diagnostic precision in assessing tumor infiltration into surrounding tissues, which can be crucial for surgical planning [2].

Surgical resection remains the cornerstone of treatment for thymic teratomas. Complete excision of the tumor is essential to alleviating the symptoms related to mass effect and to prevent recurrence. The choice of surgical approach, whether via median sternotomy or thoracotomy, depends on the tumor’s size and location [4]. In this case, a median sternotomy was performed, allowing for full access to and complete resection of the 15 x 20 cm cystic mass. Notably, the mass was adherent to the pleura and pericardium but did not invade the surrounding structures, facilitating a successful resection without the need for additional therapies.

Although chemotherapy and radiation are typically reserved for malignant teratomas, in some cases of operable tumors chemotherapy can be used preoperatively to shrink the tumor and facilitate resection [5]. The prognosis following the complete surgical resection of benign thymic teratomas is excellent, with a five-year survival rate of 95% in cases where surgery is followed by consolidation chemotherapy and radiation therapy [6].

The postoperative course for patients with mature cystic teratomas of the thymus is typically uneventful, and recurrence is exceedingly rare following complete excision. Given the location of the surgery, postoperative pulmonary complications are a significant concern. Patients should be encouraged to perform deep breathing exercises and use an incentive spirometer to prevent atelectasis and promote lung expansion. Early mobilization is crucial to enhance respiratory function and prevent complications such as pneumonia [6]. The present case report emphasizes the long-term respiratory complications of excision of a mature cystic thymus teratoma closely adherent to the pleura and pericardium which may lead to prolonged hospitalization, need for ventilatory support, and long-term chest physiotherapy. 

Regular follow-up with imaging studies is recommended to ensure there are no signs of recurrence. In this case, the patient remained asymptomatic at the one-month follow-up, consistent with the favorable long-term outlook associated with benign thymic teratomas [5,6].

Mature cystic teratomas of the thymus, although rare, require imaging and histopathology for accurate diagnosis. Surgical resection is the preferred treatment, offering an excellent prognosis with timely intervention.

## Conclusions

Mature cystic teratomas of the anterior mediastinum are exceedingly rare in 19-year-olds, highlighting the need for a detailed diagnostic approach including imaging and histopathological examination. Although these tumors are uncommon in this age group, timely surgical resection generally yields excellent outcomes. Long-term monitoring is essential to detect potential recurrence or complications early. This case underscores the importance of considering such rare conditions in differential diagnoses, the effectiveness of prompt surgical intervention in achieving favorable prognoses, and the difficult postoperative period due to complications pertaining to long-standing anterior mediastinal teratomas.
